# Exploring Non-Modifiable and Modifiable Determinants of Vision-Related Quality of Life in Central Serous Chorioretinopathy

**DOI:** 10.3390/jcm13154359

**Published:** 2024-07-25

**Authors:** Steffen E. Künzel, Payam Kabiri, Lynn zur Bonsen, Dominik P. Frentzel, Alexander Böker, Antonia M. Joussen, Oliver Zeitz

**Affiliations:** Department of Ophthalmology, Charité University Hospital Berlin, Corporate Member of Freie Universität Berlin and Humboldt-Universität zu Berlin, 10117 Berlin, Germany; payam.kabiri@charite.de (P.K.); lynn.zur-bonsen@charite.de (L.z.B.); dominik.frentzel@gmail.com (D.P.F.); alex.boeker@icloud.com (A.B.); antonia.joussen@charite.de (A.M.J.); oliver.zeitz@charite.de (O.Z.)

**Keywords:** central serous chorioretinopathy, modifiable risk factors, vision-related quality of life, sleep quality, longitudinal study

## Abstract

**Background:** To longitudinally investigate the impact of best-corrected visual acuity (BCVA), non-modifiable risk factors, modifiable habits, and disease course on the vision-related quality of life (VRQOL) of patients with central serous chorioretinopathy (CSCR). **Methods:** We longitudinally enrolled 109 CSCR patients and 42 non-diseased control participants from our clinic. In addition to clinical examination, the National Eye Institute Visual Function Questionnaire (NEI-VFQ-39) was employed for assessments, along with questions pertaining to various aspects of lifestyle habits. Alongside the cross-sectional analyses, the VRQOL of CSCR patients was tracked longitudinally over one year. **Results:** Consistent with prior studies, CSCR patients reported a lower VRQOL compared to non-diseased participants (79.3 ± 14.1 for CSCR and 92.6 ± 7.6 for CTRL; *p* < 0.0001), but fared better than those with other ocular conditions. No significant associations were observed between BCVA, any non-modifiable risk factors, or interventions, and VRQOL, both in cross-sectional and longitudinal contexts (cross-sectional BCVA with VRQOL: Pearson r correlation 0.173, *p* = 0.072). Among modifiable habits, sleep duration (*p* = 0.036), perceived quality of sleep rhythm (*p* = 0.006), hours of physical activity (*p* = 0.036), and the presence of non-ocular conditions (*p* = 0.001) were significantly correlated with VRQOL. Notably, enhanced sleep duration (+4.232 vs. −0.041 non-enhanced at 3 months, *p* = 0.033) and higher perceived quality of sleep rhythm (+6.248 vs. +0.094 non-higher, *p* = 0.009) showed a positive correlation with improved VRQOL over time. **Conclusions:** The study reveals that VRQOL has minimal dependence on BCVA or other clinical factors, suggesting that patient-reported outcome measures (PROMs) could serve as alternative endpoints in clinical studies for more holistic patient welfare assessment. Furthermore, the strong correlations between VRQOL and modifiable lifestyle habits indicate potential therapeutic value in targeting these areas for intervention.

## 1. Introduction

Central serous chorioretinopathy (CSCR) is the fourth most common non-surgical retinopathy and poses significant clinical challenges with profound implications for patients during their midlife years [1,2,3]. The condition is characterized by the accumulation of subretinal fluid (SRF) in the foveal region, pigment epithelial detachments (PED), and choroidal thickening in one or both eyes. Thus, CSCR is understood as part of the pachychoroid spectrum, a group of diseases characterized by increased choroidal thickness and hyperpermeability [4]. This spectrum includes conditions like pachychoroid pigment epitheliopathy and polypoidal choroidal vasculopathy, which share similar pathophysiological features with CSCR [4]. Thereby, the term CSCR covers two distinct entities, classically defined as the acute and chronic forms of the disease [5]. In the acute form, patients typically report symptoms such as blurred vision, relative central scotoma, metamorphopsia, moderate dyschromatopsia, hypermetropisation, micropsia, and reduced contrast sensitivity. Acute CSCR typically resolves spontaneously within 3–4 months, but can recur, with recurrence rates ranging from 15 to 50% [1,5]. Chronic CSCR is recognized by widespread tracks of RPE atrophy, characterized by their decreased fundus auto-fluorescence, and can lead to permanent symptoms including visual loss, photoreceptor degeneration, and neuroretinal atrophy [4,5]. General clinical characteristics of chronicity include significant multifocal areas of atrophic RPE alterations and/or multifocal areas of leakage. Multifocal CSCR may be more often associated with corticosteroid use [1,5]. Complications of CSCR can include fibrin deposition, which can lead to increased scar formation and severe visual loss, and a subset of patients with chronic CSCR can develop choroidal neovascularization (CNV) [4,5].

Although many patients experience spontaneous resolution, a subset has persistent disease activity, recurrent episodes, or visual symptoms reported even without active disease morphological signs [1,2]. Despite the common mild to moderate reduction in best-corrected visual acuity (BCVA) associated with CSCR, affected individuals often describe significant functional impairment in daily and professional activities, as measured by patient-reported outcome measures (PROMs) [6,7,8,9]. A number of studies suggest that BCVA, an endpoint recognized by the U.S. Food and Drug Administration (FDA) [10], might not correlate consistently with most dimensions of VRQOL in CSCR [6,9].

Both the FDA and the European Medicines Agency (EMA) advocate for the use of PROMs as complementary endpoints in clinical research [10,11,12]. One method of incorporating PROMs in ophthalmological research is through the use of standardized questionnaires [13]. The National Eye Institute Visual Function Questionnaire-39 (NEI-VFQ-39) is designed to evaluate patients’ perceptions of VRQOL and is a reputable instrument for integrating patient-reported outcomes in clinical trials. This questionnaire encompasses 12 subscales and a composite score to thoroughly evaluate various aspects of vision-related quality of life [14]. Notably, the NEI-VFQ-39 and related scoring systems have been utilized in CSCR research, indicating compromised VRQOL in the affected population and serving as an endpoint in seminal interventional studies [6,7,8,9]. 

Current therapeutic strategies for CSCR are limited in their efficacy, as systemic pharmacological treatments often exhibit side effects without clear superiority over conservative management [15,16]. Recent evidence indicates a lack of ongoing patient enrollment in CSCR drug intervention trials [17]. As a result, prevailing guidelines from leading scientific organizations do not endorse the use of systemic drugs in CSCR [18]. An alternative approach focuses on optimizing modifiable risk factors that influence disease presentation, especially as tertiary prevention for patients already diagnosed. Beyond inherent risk factors such as gender, age, genetic markers, and ethnicity, CSCR onset has been linked to several adjustable habits and external factors, including stress, type A personality, smoking, hormonal treatments, Cushing’s Syndrome, gastrointestinal conditions, sleep patterns, and others [1,4,5,16]. We have attached a detailed table of modifiable and non-modifiable habits as Supplementary Table S1 from the literature reviews [1,4,5,19,20]. Recent research has shown that CSCR patients have significantly higher stress levels at baseline compared to healthy controls, though these levels decrease over time and do not significantly differ from patients with branch retinal vein occlusion (BRVO). This suggests that stress may be a consequence of visual deterioration rather than a cause [21]. Current treatment recommendations emphasize modifying these factors, particularly stress management [18]. However, their true therapeutic potential remains under-investigated, and it is crucial to identify which factors, when adjusted, can alter disease progression.

This study sought to longitudinally assess the influence of modifiable habits on CSCR disease perception across a diverse cohort of 109 patients, spanning various disease stages, alongside a control group of 42 individuals without the disease. We intentionally curated a broad cohort of any CSCR stage with the primary interest of uncovering the relationships between adjustable habits, therapeutic interventions, and VRQOL, aiming to determine optimal conditions for patient management. Surprisingly, neither non-modifiable habits nor clinical parameters, interventions, or BCVA significantly featured on VRQOL; instead, the duration and self-reported quality of sleep emerged as significant habits strongly correlating with VRQOL. While our findings provide a foundation for patient-focused interventions, subsequent interventional research is essential to confirm efficacy.

## 2. Methods

### 2.1. Study Design

This study was part of a longitudinal, observational study prospectively recruiting patients of all CSCR disease stages as well as non-diseased participants at Charité University Hospital, Berlin, Germany. The research protocol was conducted in accordance with the valid versions of the study protocol, ICH Good Clinical Practice Guidelines (ICH-GCP), and the tenets of the Declaration of Helsinki, and was approved by the competent ethics committee of the Charité University Hospital, Berlin. All included participants provided written informed consent and were recruited prospectively.

### 2.2. Study Protocol and Subject Recruitment

From July 2021 through January 2023, affected participants who met the inclusion criteria were considered for enrollment during their regular appointments at the Department of Ophthalmology at Campus Benjamin Franklin (CBF) of Charité University Hospital, Berlin. Eligibility criteria for affected participants required participants to be adults (≥18 years) with a confirmed CSCR diagnosis. Participants provided signed informed consent, and were willing and able to attend all study visits and undergo required examinations. Initial screening included a comprehensive ocular examination with fluorescein and indocyanine green angiography, enhanced depth imaging optical coherence tomography, and fundus photography. Confirmation of CSCR was based on the presence of leakage and retinal pigment epithelium changes, subretinal fluid in OCT, or other recognized clinical indicators. Patient medical histories were thoroughly reviewed to rule out differential diagnoses and assess disease stage and progression. Exclusion criteria included: comorbid retinal diseases other than CSCR; history of retinal surgeries (exceptions: laser/photodynamic therapy, PDT); media opacities obscuring retinal visibility; and any affiliation with the study team, sponsor, or residency in the regulated institutions.

Before the start of the study, potential participants were presented with detailed information about the study’s objectives, methods, potential risks, and benefits. Only those who provided written informed consent were enrolled. In addition to the multimodal imaging, patients were required to complete a modified questionnaire, which incorporated the NEI-VFQ-39 and several other queries designed to gather more detailed information about general health, risk factors, habits, and other relevant factors. If a patient was unable to complete the questionnaire independently, an examined nurse assisted in filling it out, ensuring this was completed in a secured room to maintain patient privacy. This questionnaire (in German language) is provided as Supplementary Material File S1 attached to this article. Patients were then scheduled for regular follow-up visits to monitor the disease’s progression and assess the impact of any interventions or changes in modifiable habits. These follow-up examinations included a detailed clinical assessment as well as the completion of the questionnaire again. It is important to note that the scheduling of follow-up appointments was based on clinical necessity, not exclusively for participation in this study. Subsequent study inclusion was only considered for the study if they occurred after 3 months (±2 weeks) or a multiple thereof (e.g., 3, 6, 9, or 12 months). To uphold the highest standards of research, all clinical findings, imaging results, and patient-reported outcomes were meticulously recorded in a secure electronic database. The interdisciplinary team behind the study, which included ophthalmologists, imaging specialists, and research coordinators, regularly met to ensure a comprehensive approach to patient care and research outcomes.

The protocol, as well as inclusion and exclusion criteria for control participants, differed from those with CSCR as follows: Individuals who entered our clinic as companions in the emergency room and verbally stated that they were not suffering from CSCR were encouraged to complete the questionnaire. These individuals were added to the study following a separate ethical approval and were screened for age and gender to identify a matched cohort. No ophthalmological examination was conducted for them. Otherwise, the same inclusion and exclusion criteria as outlined above were applied.

### 2.3. Statistical Approach

Statistical analyses from raw data to final figures were conducted using Prism GraphPad version 10.2.2 and R version 4.4.0 (Prism GraphPad (GraphPad Software, San Diego, CA, USA); R (The R Foundation for Statistical Computing, Vienna, Austria). Statistical significance was established at *p* < 0.05, unless specified otherwise. For epidemiological data, a median and interquartile range (IQR) were presented, whereas figures generally depicted mean and standard error of the mean (SEM), with exceptions noted as appropriate. The Mann–Whitney U test, the Two-Tailed Fisher’s Exact Test, and the Pearson correlation were utilized for comparisons between categorical–numerical, categorical–categorical, and numerical–numerical variables, respectively. A power analysis was derived from the BIOCHOR study complex on CSCR, providing a solid basis for sample size determination. The Benjamini–Hochberg FDR correction was employed to control for multiple testing, ensuring that the probability of type I errors was minimized across the dataset. Statistical assumptions for each test were checked for adherence, and model fit was evaluated where necessary. Post-hoc tests were performed following any significant findings, and effect sizes with confidence intervals were calculated to estimate the effect magnitude and precision. To facilitate the reproducibility of our research, analysis scripts and raw data can be obtained from the corresponding author upon reasonable request. The specific statistical tests used and the color-coding guidelines for figures are explicitly stated in the figure legends.

## 3. Results

### 3.1. Cohort Characteristics

We enrolled 109 CSCR patients (154 affected eyes; 24 females) in our study based on predefined inclusion and exclusion criteria, alongside 42 age- and gender-matched controls (CTRL; 9 females). Female patients (median: 57 years old, IQR: 52.1, 64.7) were older than their male counterparts (46 with IQR: 40, 54.1, *p* < 0.05), yet they had a shorter duration since initial diagnosis (308 days [12.5, 981] for female versus 527 [124, 1755] for male patients (*p* < 0.05)), and slightly less frequently exhibited bilateral disease involvement (33.33% of female patients versus 43.53% of males, ns). The average number of visits was 2 (SD: 1.29) for male and 2.21 (SD: 1.25) for female CSCR participants. CTRL participants only completed the questionnaire, did not undergo ophthalmologic examination, and were not invited for follow-up visits. Baseline epidemiological characteristics revealed no significant differences between the CSCR patients and the CTRL group with 22% of CSCR participants being female (21.4% female CTRL), and only slight age differences without statistical significance (Table 1). Collected baseline epidemiological data are summarized in Table 1 and generally correspond with cohorts from the literature [1,2].

### 3.2. Comparative Analysis of Anamnestic Risk Habits and Lifestyle Factors between CSCR Patients and Control Participants

**Figure 1:**
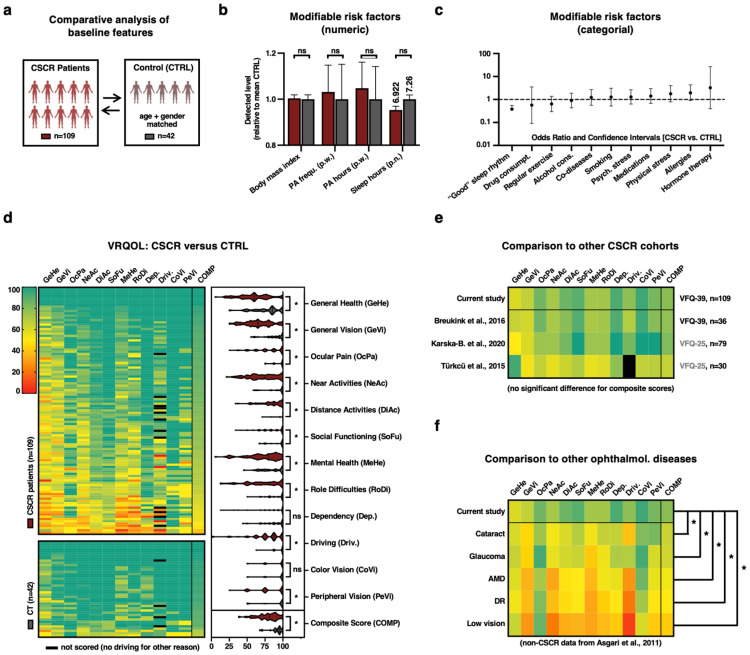
Comparative, cross-sectional analysis of CSCR patients and non-diseased controls. (**a**). Overview: Baseline parameters from 109 CSCR patients juxtaposed with 42 non-diseased controls. (**b**,**c**). Distinctive comparison of numeric in b and categorical in c risk factors between the two groups. The control group’s mean is standardized to 1. Color-coding: CSCR group: red, versus control group: grey. Error bars represent the standard error of the mean (s.e.m.). Statistical significance indicated by * (*p* < 0.05) determined using the Mann–Whitney U test. Categorical attributes display odds ratios, with the Two-Tailed Fisher’s Exact Test applied to c (for consistency reasons for all categorial 11 features). Benjamini–Hochberg FDR correction for all *p*-values of b and c combined. (**d**). Left panel: A heatmap showcasing VRQOL distribution among CSCR patients (upper section) and CTRL (lower section). Refer to the top-left color scale for intensity indication. Right panel: Violin plots elucidating the variations across the 12 NEI-VFQ-39 subscales and the overarching score between the CSCR and control groups at the bottom. Significance levels marked by * (*p* < 0.05) based on the Mann–Whitney U test (FDR-corrected). (**e**). Comparative evaluation of VRQOL scores in the present research against prior studies on CSCR, revealing no marked differences [6,7,8]. (**f**). Contrast of VRQOL scores between CSCR patients and common ocular conditions as documented in [22]. A two-sided *t*-test was employed for both (**e**,**f**).

In our baseline analysis comparing anamnestic habits between CSCR and CTRL cohorts (Figure 1a–c), we found no significant difference for numerical variables like body mass index (BMI; CSCR: 25.55 ± 4.07 vs. CTRL: 25.39 ± 3.09; *p* = 0.352) and frequency of physical activity (PA; times per week; CSCR: 1.4 ± 1.61 vs. CTRL: 1.44 ± 1.42; *p* = 0.8596). This also applied to hours of PA per week (CSCR: 1.53 ± 1.73 vs. CTRL: 1.46 ± 1.35; *p* = 0.8596). A notable difference emerged regarding hours of sleep per night (CSCR: 6.922 ± 1.22 vs. 7.26 ± 0.86, *p* = 0.352; non-FDR-corrected *p*-value = 0.0469; Figure 1b) with CSCR patients sleeping on average about 20 min less per night than CTRL participants. As for categorical variables (Figure 1c), CTRL individuals more often described their sleep rhythm as “good” (29/42) compared to CSCR patients (50/109, OR: 0.379, *p* = 0.174, non-FDR-corrected *p* = 0.0116). However, this was a non-significant post-strict FDR correction. CSCR patients had a slightly higher likelihood of previous hormone therapy, yet this was again not significant (8/109 vs. 1/41; OR = 3.25; *p* = 0.8596; Figure 1c). Other categorical variables, like descriptions of physical stress (OR = 1.783, *p* = 0.672), psychological stress (OR = 1.293, *p* = 0.8596), regular physical activity (OR = 0.638, *p* = 0.344), smoking (OR = 1.265, *p* = 0.666), alcohol consumption (OR = 0.905, *p* = 0.8596), medication (OR = 1.416, *p* = 0.8596), allergies (OR = 1.951, *p* = 0.47), co-diseases (OR = 1.233, *p* = 0.8596), and drug abuse (OR = 0.566, *p* = 0.8596), showed no significant differences between CSCR and CTRL participants (all *p*-values FDR-corrected).

### 3.3. Comparative Analysis of VRQOL Subscale Scores between CSCR Patients and Non-Diseased CTRL Group

We assessed the VRQOL by comparing scores across all subscales between CSCR and CTRL participants (Figure 1d and Supplementary Figure S1). Our findings revealed significant disparities in almost all VRQOL dimensions: Participants with CSCR reported lower scores in General Health (GeHe), with an average of 59.9 compared to 78.6 in controls, which was statistically significant (*p* < 0.0001). A similar trend was observed in the General Vision (GeVi) subscale, where CSCR participants scored 66.2 against controls’ 91.1 (*p* < 0.0001). Ocular Pain (OcPa) also differed markedly, with scores of 84.4 for CSCR and 96.1 for controls (*p* < 0.0001). Near Activities (NeAc) and Distant Activities (DiAc) were other areas where CSCR patients reported lower quality of life scores, at 72.5 and 85.6, respectively, versus 95.1 and 96.5 in the control group (*p* < 0.0001 for both). Social Functioning (SoFu) scores were slightly closer, yet still significantly different, at 89.5 for CSCR and 95.4 for controls (*p* = 0.013). In terms of Mental Health (MeHe), CSCR participants’ scores were also lower, averaging 72.9 compared to 86.8 for the control group (*p* < 0.0001). Role Difficulties (RoDi) presented a similar pattern, with CSCR participants scoring 73.7 against controls’ 93.6 (*p* < 0.0001). Driving abilities (Driv.) were also affected, with scores of 78.2 for CSCR participants and 97 for controls (*p* < 0.0001). Lastly, in the Peripheral Vision (PeVi) category, CSCR patients scored 83.3, lower than controls’ 94.6 (*p* = 0.0003). Consequently, we identified a statistical difference in 10/12 subscales and the composite score (COMP) between the two groups: 79.3 ± 14.1 for CSCR and 92.6 ± 7.6 for CTRL (*p* < 0.0001). Notably, the subscores for Dependency (Dep.) and Color Vision (CoVi) did not show statistical differences between the cohorts, with scores of 91.5 ± 17.5 for CSCR vs. 90.6 ± 14.1 for CTRL (*p* = 0.215), and 94.3 ± 13 for CSCR vs. 95.8 ± 13.3 for CTRL (*p* = 0.4306), respectively.

### 3.4. VRQOL Comparison of Our CSCR Cohort Versus the Published Literature and Other Ocular Diseases

Next, we compared our cohort’s VRQOL with studies that already focused on VRQOL in CSCR [6,7,8]. Although there were differences in sample size, inclusion criteria, and VFQ-test versions among these studies, no statistical difference was observed between our CSCR group’s composite scores and those of other CSCR patient groups (Figure 1e). Furthermore, our CSCR cohort’s VRQOL composite score (79.3 ± 14.1) was significantly higher than those of Cataract (64.56 ± 3.71), Glaucoma (62.96 ± 6.35), ARMD (53.07 ± 6.91), Diabetic Retinopathy (50.53 ± 6.01), and other Low Vision patients (41.90 ± 5.72; Figure 1f) [22].

### 3.5. Determinants of VRQOL in CSCR Patients: A Comprehensive Baseline Analysis

**Figure 2:**
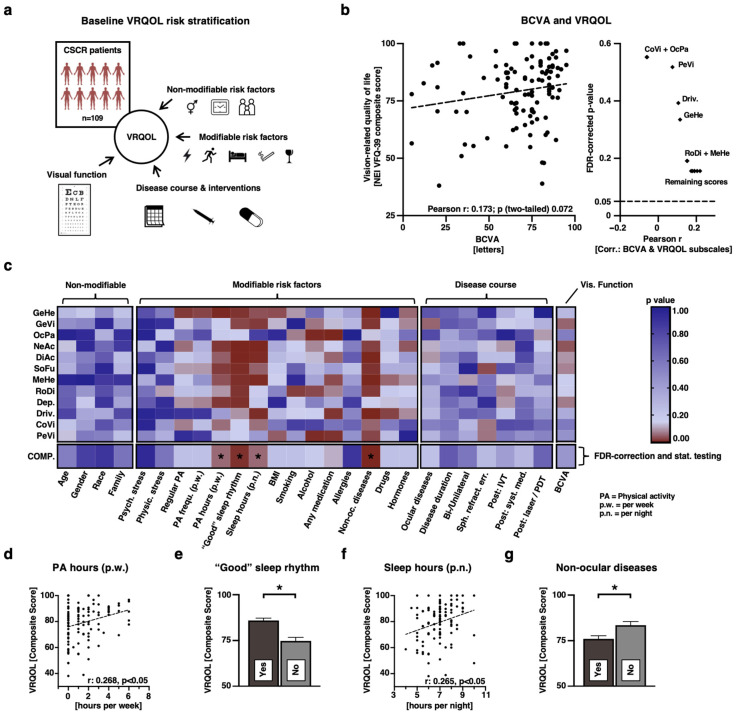
VRQOL risk stratification at baseline among CSCR patients. (**a**). Overview: The investigation analyzed the association between CSCR patients’ VRQOL and factors including visual function, non-modifiable and modifiable risk elements, as well as disease progression and therapeutic interventions. (**b**). Left panel: Scatter plot contrasting the NEI-VFQ-39 composite score with BCVA. Analysis: Pearson’s r, two-tailed *p*-value testing, no FDR correction. Right panel: Corresponding *p*-values for NEI-VFQ-39 subscale scores against BCVA. This analysis mirrors the method employed for the composite score on the left. FDR correction was applied. (**c**). Representation of *p*-values derived from examining all 12 subscales and the composite score against all logged features categorized into non-modifiable, modifiable, disease progression and interventions, and visual acuity. Notably, *p*-values are not individually adjusted except for the composite score (FDR correction). Color scale at the right. Pearson’s r and two-tailed *p*-values were deduced for numeric attributes, while the Mann–Whitney U test was utilized for categorical features. Statistical significance is marked by * (*p* < 0.05) and adjustments made using the Benjamini–Hochberg method for FDR correction. (**d**–**g**). Visual representations of the four predominant features that exhibit a significant correlation with the NEI-VFQ-39 composite score. For (**d**,**g**), Pearson correlation was applied with the metrics in the figure. For e and g, mean and S.E.M. was applied with * (*p* < 0.05).

We sought to determine the factors that influence VRQOL in CSCR patients, focusing solely on the diseased cohort (Figure 2a). Our primary assessment centered on the impact of BCVA on VRQOL (Figure 2b). Surprisingly, there was no significant correlation between the BCVA of the most affected eye and the composite score of the NEI-VFQ-39 (Pearson r: 0.173; *p* = 0.072, Figure 2b left), or its individual subscales (Figure 2b right). Nevertheless, there was a discernible trend for the composite score and certain subscales (see also Supplementary Figure S1b).

In a broader analysis, we evaluated the effects of all 27 anamnestic attributes on the 12 subscales and their composite scores (Figure 2c). Remarkably, non-modifiable risks such as age, gender, race, and family history, along with disease characteristics (e.g., ocular diseases, disease duration, treatment histories) did not exhibit a significant impact on VRQOL in CSCR. In stark contrast, several modifiable risk behaviors significantly featured on multiple VRQOL dimensions (evidenced by the red color patterns in the modifiable risk factors cluster in Figure 2c). Post FDR-correction on composite scores, four critical modifiable behaviors stood out: hours of physical activity (PA) each week (*p* = 0.036), perceived quality of sleep rhythm (*p* = 0.006), sleep duration each night (*p* = 0.036), and non-ocular health conditions (*p* = 0.001, Figure 2c–g). Specifically, individuals reported better VRQOL scores with increased weekly PA (Pearson r: 0.268), positive perceived sleep rhythm quality, longer sleep durations (r: 0.265), and fewer non-ocular health issues (Figure 2d–g).

### 3.6. Impact of Habitual Changes on VRQOL: A Longitudinal Approach

**Figure 3:**
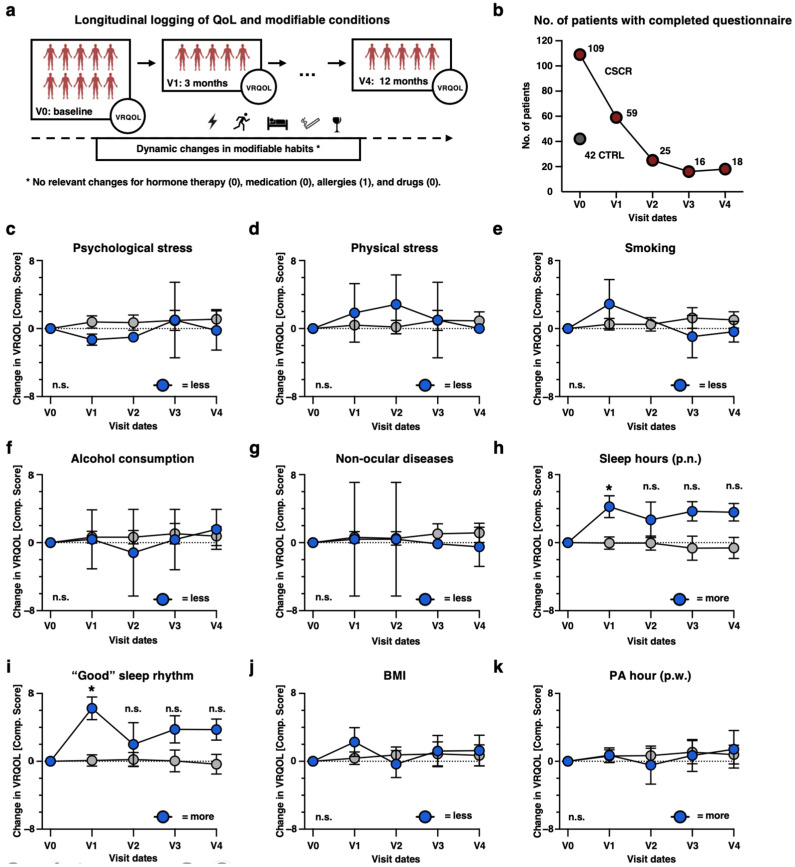
Longitudinal assessment of VRQOL among CSCR patients over time. (**a**). Overview: Changes in the NEI-VFQ-39 composite scores were analyzed in the context of alterations in patients’ modifiable habits over time. (**b**). Graph delineating the number of finalized questionnaires alongside their respective completion timepoints, distinguishing between CSCR patients (represented in red) and controls (in grey, who completed the questionnaire solely at the baseline phase). (**c**–**k**). Longitudinal comparison of VRQOL centering on the variance between patients who underwent changes in their modifiable habits against those who did not. The specific modifiable habit under consideration is indicated within the graph’s title and its direction at its bottom right corner. Mean of baseline scores for each group equals 0. Shown are changes from this over time. Mann–Whitney U test was utilized for comparison between groups. Any statistical significance is demarcated by an asterisk (*), corresponding to a *p*-value of less than 0.05, while “n.s.” denotes instances where the findings were statistically non-significant. Benjamini–Hochberg FDR correction applies.

In a comprehensive longitudinal study, we sought to determine the correlation between changes in risk habits and alterations in VRQOL over time. Participants were evaluated at designated intervals: baseline (V0), three months post-baseline (V1), three months post-V1 (V2), and so forth, culminating at a 12-month study period (Figure 3a). It is worth noting that changes in certain habits like hormone therapy, medication intake, allergies, and drug abuse were not frequently observed (Figure 3a). Owing to our study’s criteria, participants were engaged for follow-up visits based on clinical needs rather than exclusive study participation, resulting in a diminishing patient count as the study progressed (Figure 3b).

Figure 3c–k graphically illustrate the influence of modifiable habit alterations on VRQOL composite scores. Blue markers signify changes deemed favorable from literary research (e.g., in Figure 3c: reduced psychological stress), while grey markers indicate unchanged or deteriorating conditions (e.g., consistent or increased psychological stress). Intriguingly, most changes in conditions had no marked influence on VRQOL. However, an exception emerged in the realm of sleep. Participants who slept longer periods consistently recorded improved VRQOL scores over time (+4.232 vs. −0.041 at 3 months, *p* = 0.033). Moreover, those reporting enhanced perceived sleep rhythm quality showcased a significant uplift in VRQOL at 3 months (+6.248 vs. +0.094, *p* = 0.009). While we observed some trends over the long term, they did not withstand our FDR correction, likely due to the small sample size after V1.

## 4. Discussion

Our exploration concerning VRQOL in CSCR patients brought potential relationships between epidemiological features, lifestyle habits, clinical interventions, and their potential link to VRQOL to light. As main findings, we noticed that while higher BCVA is usually seen as a main goal in clinical CSCR management and a clinical endpoint in interventional trials, it does not capture how patients feel about their disease. In contrast, the duration and quality of sleep rhythm, along with physical activity and non-ocular comorbidities, stood out as general habits that are strongly statistically associated with VRQOL.

The CSCR cohort consistently exhibited significantly poorer VRQOL across all subscales compared to CTRL participants, yet better than those with other common ocular diseases (Figure 1). The weak correlation between BCVA and VRQOL depicted in Figure 2b and Supplementary Figure S1, makes clear that patients with CSCR experience significant distress even though many have clinically satisfying BCVA (Figure 2). This core finding underscores the importance of incorporating patient-reported outcome measures (PROMs) in clinical trials and disease monitoring for CSCR, and highlights the need for a broader diagnostic approach that includes patient subjective experiences to fully understand the impact of the disease.

Furthermore, our baseline VRQOL analysis presented in Figure 2 yields surprising results. Non-modifiable risk factors (such as age, gender, race, and family history), as well as numerous clinical disease courses and BCVA, had virtually no relevant correlation with VRQOL—neither with the composite score nor with individual subscales (Figure 2c). This was unexpected, as it includes key clinical parameters such as BCVA, duration of illness, unilateral or bilateral occurrence, and whether extensive interventional treatments such as intravitreal treatment (IVT) or photodynamic therapy (PDT) have been carried out (Figure 2c). In contrast, some modifiable risk habits strongly correlate with almost every VRQOL scale. Notably, the accumulated duration of weekly sports activities, as well as the duration and perceived quality of sleep rhythm, and non-ocular diseases stand out (Figure 2c). Especially, sleep habits have already been well described as factors that can trigger the disease and define its course [23,24]. Our findings suggest that affected CSCR patients in our cohort sleep about 20 min less than CTRL participants on average, perceive a lower quality of sleep rhythm (Figure 1), and that the sleep duration, as well as the perceived quality strongly correlate with almost every dimension of VRQOL (Figure 2). Moreover, patients who change their sleep habits for the better (time or quality) report a significantly higher VRQOL after 3 months (Figure 3). Another interesting finding is that CSCR patients do not report co-morbidities more frequently than the CTRL cohort (Figure 1c), yet they perceive the General Health (GeHe) subscale as significantly poorer (Figure 1d). This could be due to the perception—or the severity—of the non-ocular co-diseases that we have not captured here in more depth. For all four significant modifiable habits, we found that the expected preferred phenotype indeed correlates with higher VRQOL (Figure 2d–g). This is partially in contrast with other studies—especially about physical activity [25].

There are multiple limitations and possible biases to be acknowledged. First, all shown correlations do not imply directional causality or causality at all. It could be the case that people who sleep better and longer suffer less from the disease due to the regenerative function of sleep—but it could also be the opposite: people who suffer less from the disease might sleep better because they worry less. Or neither. To demonstrate causality, the implementation of interventional studies should be considered—which is entirely feasible through sleep improvement apps without relevant side effects to be expected, for example [26]. Second, researchers must be cautious whether the correlations shown are specific to CSCR or a general observation. For example, the notion that all people (including healthy ones) who sleep more and better might have a higher quality of life. We believe that we can partially refute this for several reasons: (a) CSCR patients in our cohort indeed sleep less and worse than non-diseased individuals. (b) The validated VRQOL is supposed to specifically reflect the VRQOL and not general quality of life. (c) In our CTRL cohort, VRQOL is not higher for people who sleep more (*p* = 0.33), sleep better (“good” sleep rhythm, *p* = 0.021), or engage in more sports (*p* = 0.88)—although it must be added that the non-diseased participants generally achieve almost solely maximal values for VRQOL, making a statistical approach difficult at this point. Third, we would like to highlight the inclusion bias in our academic setting that might not reflect the general CSCR population with potentially more complex cases. This is particularly true for the longitudinal approach, in which patients only visited the clinic when necessary, from a clinical point of view (compare methods). It lays in the very nature of this, that this creates a strong bias for more severe CSCR cases. This bias is particularly true for the examined interventions: We believe that the observation of patients with prior IVT or PDT not having higher VRQOL scores than those without such treatments (Figure 2c) does not allow us to conclude that these interventions do not affect VRQOL. Rather, these treatments were naturally performed on patients who were more severely affected by the disease from the outset. Additionally, not performing ophthalmological examinations in the control group is a limitation we acknowledge. Due to logistical constraints, it was not feasible to recall and examine the control participants. Although there is a possibility that undiagnosed cases of CSCR or other conditions within the pachychoroid spectrum may be present in the control group, we believe this likelihood is small.

In summary, our investigation into VRQOL in CSCR patients not only corroborates prior findings but also sheds light on the complex interplay between lifestyle habits and patient-perceived disease impact. It calls into question the sufficiency of traditional clinical endpoints, such as BCVA, in truly capturing patient well-being. Instead, our study illustrates the substantial role of modifiable lifestyle factors, like sleep and physical activity, in affecting VRQOL. This suggests that interventions aimed at improving these habits could enhance patient quality of life, independent of the disease’s clinical status. As such, our findings advocate for the inclusion of PROMs in the evaluation of therapeutic outcomes and in routine clinical assessments to provide a more holistic view of patient health.

## Figures and Tables

**Table 1:** Baseline Characteristics of CSCR Patients and Healthy Controls.

	CSCR Male	CSCR Female	Healthy Control
Patients (affected eyes)	85 (122)	24 (32)	42 [33 male/9 female]
Age in years (median [IQR])	46 [40, 54.1]	57 [52.1, 64.7]	49 [42.1, 55.9]
Days since initial diagnosis (median [IQR])	527 [124, 1755]	308 [12.5, 981]	n.a.
Currently active disease (patients)	71 (83.53%)	20 (83.33%)	n.a.
Bilateral disease (patients)	37 (43.53%)	8 (33.33%)	n.a.
Post PDT (patients)	6 (7.06%)	0 (0%)	n.a.
Post laser surgery (patients)	8 (9.41%)	4 (16.67%)	n.a.
Post systemic treatment (patients)	32 (37.65%)	4 (16.67%)	n.a.
Best-corrected visual acuity in Log-MAR (median [IQR])	75 [63, 84]	65.5 [44.25, 75]	n.a.
Number of logged visits (mean [SD])	2 [1.29]	2.21 [1.25]	1 [0]

## Data Availability

The original contributions presented in the study are included in the article/Supplementary Material, further inquiries can be directed to the corresponding author.

## Supplementary Materials

The following supporting information can be downloaded at: https://www.mdpi.com/article/10.3390/jcm13154359/s1, Figure S1: NEI-VFQ39 scores between CSCR patients and non-diseased probands, and correlation of BCVA with NEI-VFQ39 subscales. Table S1: Risk factors for CSCR from literature.
