# Supplementary material for: Exploring Non-Modifiable and Modifiable Determinants of Vision-Related Quality of Life in Central Serous Chorioretinopathy
Source: J Clin Med. 2024 Jul 25;13(15):4359. doi: 10.3390/jcm13154359 (PMC11313338; doi:10.3390/jcm13154359)
Supplement: Supplementary file 1 [file jcm-13-04359-s001.zip › Suppl. Figure S1.pdf]

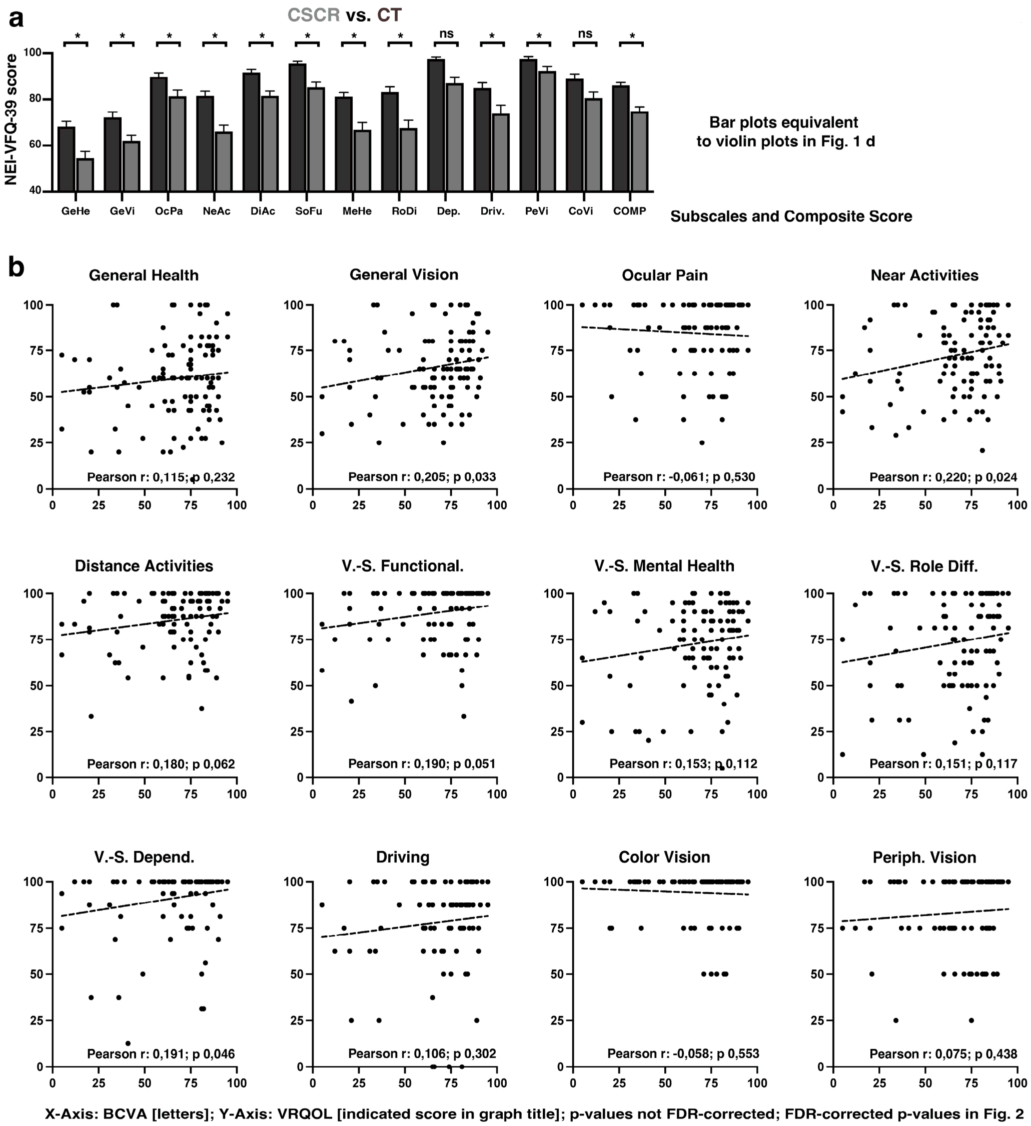

Figure S1: NEI-VFQ39 scores between CSCR patients and non-diseased probands (a), and correlation of BCVA with NEI-VFQ39 subscales (b).
