# Supplementary material for: Exploring Non-Modifiable and Modifiable Determinants of Vision-Related Quality of Life in Central Serous Chorioretinopathy
Source: J Clin Med. 2024 Jul 25;13(15):4359. doi: 10.3390/jcm13154359 (PMC11313338; doi:10.3390/jcm13154359)
Supplement: Supplementary file 1 [file jcm-13-04359-s001.zip › Suppl. Material S1 _ Questionnaire_VRQOL_CSCR_REVISED.pdf]

CharitéCentrum für Audiologie und Phoniatrie, Augen- und HNO-Heilkunde

Charité Augenklinik | Campus Benjamin Franklin | 12203 Berlin

Augenklinik und Poliklinik
Direktorin: Univ.-Prof. Dr. med. Antonia Joussen

Campus Benjamin Franklin
Hindenburgdamm 30, 12203 Berlin
Tel. +49 30 8445 2331
Fax.+49 30 8445 4450

Liebe/r Studienteilnehmer/in,

an dieser Stelle ein herzliches Dankeschön, dass Sie sich für die Teilnahme an dieser Studie entschieden haben. Diese beinhaltet u.a. das Ausfüllen des nachfolgenden Fragebogens. Der Fragebogen besteht aus 69 Fragen zu verschiedenen Facetten der Chorioretinopathia Centralis Serosa. Diese wird im nachfolgenden abgekürzt CSCR genannt. Die Fragen können schnell ohne ein längeres Nachdenken beantwortet werden.

Sollten Sie Fragen haben, können Sie diese jetzt und jederzeit stellen oder alternativ an steffen-emil.kuenzel@charite.de. Bei Problemen beim Ausfüllen des Fragebogens, bieten wir Ihnen gerne unsere Unterstützung an.

Ihre Antworten werden – wie alle persönlichen Informationen, die im Rahmen dieser Studie erhoben wurden und werden – streng vertraulich behandelt und nicht an Dritte weitergegeben.

Herzlichen Dank,
Ihr Studienteam

Zu Beginn möchten wir Ihnen 26 Fragen zu möglichen Auslösern Ihrer Erkrankung stellen?

1.	Spüren Sie **aktuell körperlichen Stress**?	Ja	Nein
2.	Haben Sie **bei Krankheitsbeginn** der CSCR **körperlichen Stress** verspürt?	Ja	Nein
3.	Spüren Sie **aktuell psychischen Stress**?	Ja	Nein
4.	Haben Sie **bei Krankheitsbeginn** der CSCR **psychischen Stress** verspürt?	Ja	Nein
5.	Sind sie **aktuell schwanger**?	Ja	Nein
6.	Waren Sie **bei Krankheitsbeginn** der CSCR **schwanger**?	Ja	Nein
7.	**Rauchen** Sie aktuell?	Ja	Nein
8.	Haben Sie **bei Krankheitsbeginn** der CSCR **geraucht**?	Ja	Nein
9.	Trinken Sie **aktuell** regelmäßig **Alkohol**? Wenn ja, wieviel und wie häufig? ______________________________________	Ja	Nein
10.	Haben Sie **bei Krankheitsbeginn** der CSCR regelmäßig **Alkohol** getrunken? Wenn ja, wieviel und wie oft? ______________________________________	Ja	Nein
11.	Nehmen Sie **aktuell Medikamente** ein? Wenn ja, welche? ______________________________________	Ja	Nein
12.	Haben Sie **bei Krankheitsbeginn** der CSCR **Medikamente** ein? Wenn ja, welche? ______________________________________	Ja	Nein
13.	Leiden Sie **aktuell** an **anderen Erkrankungen** außer der CSCR? Wenn ja, welche? ______________________________________	Ja	Nein
14.	Haben Sie **bei Krankheitsbeginn** der CSCR an **anderen Erkrankungen** gelitten? Wenn ja, welche? ______________________________________	Ja	Nein
15.	Nehmen Sie **aktuell** regelmäßig **Drogen** ein? Wenn ja, welche und wie häufig? ______________________________________	Ja	Nein
16.	Nahmen Sie **bei Krankheitsbeginn** der CSCR regelmäßig **Drogen** ein? Wenn ja, welche und wie häufig? ______________________________________	Ja	Nein
17.	Wieviele Stunden **schlafen** Sie aktuell pro Nacht?	_____ h	
18.	Würden Sie Ihren **Schlafrhythmus** als gut bezeichnen?	Ja	Nein
19.	Hat sich Ihr **Schlafrhythmus seit Krankheitsbeginn** der CSCR verändert? Falls ja, inwiefern? ______________________________________	Ja	Nein
20.	Haben Sie sich in Ihrem Leben bereits einer **Hormontherapie** unterzogen? Wenn ja, welche und wann? ______________________________________	Ja	Nein
21.	Haben Sie **Allergien**? Wenn ja, welche? ______________________________________	Ja	Nein

22.	Wie **groß** sind Sie?	_____ cm	
23.	Wie **schwer** sind Sie (als Sie sich zuletzt gewogen haben)?	_____ kg	
24.	Wie oft in der Woche machen Sie **Sport**? Wieviele Stunden circa?	_____ mal<br>_____ h	
25.	Gibt es in **Ihrer Familie andere Personen** mit CSCR? Wenn ja, wer?<br>___________________________________	Ja	Nein
26.	Denken Sie, dass die bei Ihnen diagnostizierte Erkrankung der CSCR in Zusammenhang mit einem anderen, hier nicht aufgeführten, **Auslöser** steht? Wenn ja, welcher?<br>___________________________________	Ja	Nein

Nun geht es weiter mit allgemeinen Fragen zur Krankheit, ihren Symptomen und mögliche Lebenseinschränkungen.

27. Wie würden Sie im allgemeinen Ihren Gesundheitszustand beschreiben?

Ausgezeichnet	Sehr Gut	Gut	Mittelmäßig	Schlecht
1	2	3	4	5

28. Wie würden Sie heute Ihre Sehkraft bewerten, die sie mit Brille oder Kontaktlinsen haben?

Ausgezeichnet	Gut	Mittelmäßig	Schlecht	Sehr schlecht	Vollständig blind
1	2	3	4	5	6

29. Wie oft sorgen Sie sich um Ihre Sehkraft?

Niemals	Selten	Manchmal	Meistens	Immer
1	2	3	4	5

30. In welchem Ausmaß hatten Sie in der Vergangenheit Schmerzen oder Beschwerden in und um Ihre Augen?

Gar keine	Leichte	Mittelmäßige	Starke	Sehr starke
1	2	3	4	5

31. Wie groß sind Ihre Schwierigkeiten beim Lesen einer normal gedruckten Zeitung?

Keine	Wenig	Ziemlich	Stark	Wegen Sehkraft aufgehört	Aus anderen Gründen aufgehört oder kein Interesse
1	2	3	4	5	6

32. Wie stark sind Ihre Schwierigkeiten, wenn Sie auf Ihrer Arbeit oder beim Ausüben von Hobbies in der Nähe gut sehen müssen, z.B. beim Kochen, Nähen, bei der Hausarbeit oder beim Benutzen von Werkzeug. Würden Sie sagen:

Keine	Wenig	Ziemlich	Stark	Wegen Sehkraft aufgehört	Aus anderen Gründen aufgehört oder kein Interesse
1	2	3	4	5	6

33. Haben Sie aufgrund Ihrer Sehkraft Probleme, etwas in einem unübersichtlichen Regal zu finden?

Keine	Wenig	Ziemlich	Stark	Wegen Sehkraft aufgehört	Aus anderen Gründen aufgehört oder kein Interesse
1	2	3	4	5	6

34. Wie groß sind Ihre Schwierigkeiten, Straßenschilder oder die Namen von Geschäften zu lesen?

Keine	Wenig	Ziemlich	Stark	Wegen Sehkraft aufgehört	Aus anderen Gründen aufgehört oder kein Interesse
1	2	3	4	5	6

35. Haben Sie aufgrund Ihrer Sehkraft bei Dämmerung oder in der Nacht Schwierigkeiten, Stufen, Treppen oder den Bordstein herabzusteigen?

Keine	Wenig	Ziemlich	Stark	Wegen Sehkraft aufgehört	Aus anderen Gründen aufgehört oder kein Interesse
1	2	3	4	5	6

36. Haben Sie aufgrund Ihrer Sehkraft Schwierigkeiten, Dinge neben sich zu sehen, wenn Sie irgendwo entlang gehen?

Keine	Wenig	Ziemlich	Stark	Wegen Sehkraft aufgehört	Aus anderen Gründen aufgehört oder kein Interesse
1	2	3	4	5	6

37. Haben Sie aufgrund Ihrer Sehkraft Schwierigkeiten zu sehen, wie andere auf das reagieren, was Sie gerade gesagt haben?

Keine	Wenig	Ziemlich	Stark	Wegen Sehkraft aufgehört	Aus anderen Gründen aufgehört oder kein Interesse
1	2	3	4	5	6

38. Haben Sie aufgrund Ihrer Sehkraft Schwierigkeiten, Ihre eigene Kleidung farblich zusammenzustellen?

Keine	Wenig	Ziemlich	Stark	Wegen Sehkraft aufgehört	Aus anderen Gründen aufgehört oder kein Interesse
1	2	3	4	5	6

39. Habe Sie aufgrund Ihrer Sehkraft Schwierigkeiten, neue Bekannte in deren Wohnung zu besuchen oder auf Partys oder im Restaurant zu erkennen?

Keine	Wenig	Ziemlich	Stark	Wegen Sehkraft aufgehört	Aus anderen Gründen aufgehört oder kein Interesse
1	2	3	4	5	6

40. Haben Sie aufgrund Ihrer Sehkraft Schwierigkeiten, ins Kino oder Theater zu gehen oder Sportveranstaltungen zu besuchen?

Keine	Wenig	Ziemlich	Stark	Wegen Sehkraft aufgehört	Aus anderen Gründen aufgehört oder kein Interesse
1	2	3	4	5	6

Nun kommen Fragen zum Autofahren.

41. Autofahren: Fahren Sie zur Zeit regelmäßig oder wenigstens manchmal Auto?

Ja	Nein
1	2

42. Autofahren: Wenn nein. Sind Sie bisher nie Auto gefahren oder haben Sie das Auto fahren aufgegeben?

Bin nie Auto gefahren	Habe das Auto fahren aufgegeben
1	2

43. Autofahren: Wenn Sie das Autofahren aufgegeben haben. War es hauptsächlich wegen der Sehkraft, gab es andere Gründe, oder war es sowohl wegen Ihrer Sehkraft als auch aus anderen Gründen?

Hauptsächlich wegen Sehkraft	Hauptsächlich wegen anderer Gründe	Sowohl wegen der Sehkraft als auch aus anderen Gründen
1	2	3

44. Autofahren. Wie starke Schwierigkeiten haben Sie beim Autofahren, wenn Sie am Tag durch bekannte Gegenden fahren?

Keine	Wenig	Ziemlich	Starke
1	2	3	4

45. Autofahren: Haben Sie aufgrund Ihrer Sehkraft Schwierigkeiten, nachts Auto zu fahren?

Keine	Wenig	Ziemlich	Stark	Wegen Sehkraft aufgehört	Aus anderen Gründen aufgehört oder kein Interesse
1	2	3	4	5	6

Die nächsten Fragen behandeln Dinge, die Sie vielleicht aufgrund Ihrer Sehkraft tun, z.B. bei der Arbeit oder bei täglichen Aktivitäten im Haushalt, bei der Kinderpflege, in der Schule oder bei gesellschaftlichen Aktivitäten. Bei jeder Frage antworten Sie bitte ob dies für Sie immer, meistens, manchmal, selten oder nie zutrifft.

Immer	Meistens	Manchmal	Selten	Nie
1	2	3	4	5

46.	Bringen Sie weniger zustande, als Sie sich vorgenommen haben?	1	2	3	4	5
47.	Können Sie nicht so lange arbeiten oder andere Aktivitäten durchführen wie Sie gerne möchten?	1	2	3	4	5

48. Wie stark hindern Sie Schmerzen oder Beschwerden in und um Ihre Augen, wie z.B. Brennen der Augen, Hucken, Schmerzen, daran, das zu tun, was Sie tun möchten? Würden Sie sagen:

Niemals	Manchmal	Die Hälfte	Die meiste Zeit	Immer
1	2	3	4	5

Die nächsten Fragen behandeln Ihr Umgehen mit Ihrem Sehvermögen. Bitte markieren Sie für jede Aussage, ob sie für Sie absolut richtig, meistens richtig, nicht sicher, meistens falsch oder absolut falsch ist.

Absolut richtig	Meistens richtig	Weiß nicht	Meistens falsch	Absolut falsch
1	2	3	4	5

49.	Wegen meiner Sehkraft bleibe ich die meiste Zeit zu Hause.	1	2	3	4	5
50.	Wegen meiner Sehkraft bin ich die meiste Zeit gehemmt.	1	2	3	4	5
51.	Wegen meiner Sehkraft habe ich sehr wenig Kontrolle über das, was ich tue.	1	2	3	4	5
52.	Wegen meiner Sehkraft muss ich mich zu sehr auf andere Menschen verlassen.	1	2	3	4	5
53.	Wegen meiner Sehkraft benötige ich viel Hilfe von anderen.	1	2	3	4	5

Bei der folgenden Aussage markieren Sie bitte, ob sie für Sie absolut richtig, meistens richtig, nicht sicher, meistens falsch oder absolut falsch ist.

Absolut richtig	Meistens richtig	Weiß nicht	Meistens falsch	Absolut falsch
1	2	3	4	5

54.	Ich mache mir Sorgen darüber, dass ich wegen meiner eingeschränkten Sehkraft mir oder anderen Probleme bereiten werde.	1	2	3	4	5

55. Wir würden Sie Ihren allgemeinen Gesundheitszustand auf einer Skala bewerten, bei der 0 die schlechteste mögliche und 10 die bestmögliche Gesundheit bezeichnet?

Schlechteste									Beste
1	2	3	4	5	6	7	8	9	10

56. Wir würden Sie Ihre heutige Sehkraft auf einer Skala bewerten, bei der 0 die schlechteste mögliche und 10 die bestmögliche Gesundheit bezeichnet?

Schlechteste									Beste
1	2	3	4	5	6	7	8	9	10

57. Wenn Sie Ihre Brille oder Kontaktlinsen tragen, wie stark sind Ihre Schwierigkeiten, wenn Sie die kleine Schrift im Telefonbuch, auf einer Arzneiflasche oder auf einem Formular lesen wollen?

Keine	Wenig	Ziemlich	Stark	Wegen Sehkraft aufgehört	Aus anderen Gründen aufgehört oder kein Interesse
1	2	3	4	5	6

58. Haben Sie aufgrund Ihrer Sehkraft Schwierigkeiten herauszufinden, ob die Rechnung, die man Ihnen ausstellt, korrekt ist?

Keine	Wenig	Ziemlich	Stark	Wegen Sehkraft aufgehört	Aus anderen Gründen aufgehört oder kein Interesse
1	2	3	4	5	6

59. Haben Sie aufgrund Ihrer Sehkraft Schwierigkeiten, sich vor dem Spiegel zu rasieren oder Ihr Haar zu frisieren, oder sich zu schminken?

Keine	Wenig	Ziemlich	Stark	Wegen Sehkraft aufgehört	Aus anderen Gründen aufgehört oder kein Interesse
1	2	3	4	5	6

60. Haben Sie aufgrund Ihrer Sehkraft Schwierigkeiten, Menschen, die Sie kennen, in einem Raum wiederzuerkennen?

Keine	Wenig	Ziemlich	Stark	Wegen Sehkraft aufgehört	Aus anderen Gründen aufgehört oder kein Interesse
1	2	3	4	5	6

61. Haben Sie aufgrund Ihrer Sehkraft Schwierigkeiten zu joggen oder spazieren zu gehen?

Keine	Wenig	Ziemlich	Stark	Wegen Sehkraft aufgehört	Aus anderen Gründen aufgehört oder kein Interesse
1	2	3	4	5	6

62. Haben Sie aufgrund Ihrer Sehkraft Schwierigkeiten, das Fernsehprogramm zu sehen und sich daran zu erfreuen?

Keine	Wenig	Ziemlich	Stark	Wegen Sehkraft aufgehört	Aus anderen Gründen aufgehört oder kein Interesse
1	2	3	4	5	6

63. Haben Sie aufgrund Ihrer Sehkraft Schwierigkeiten, Freunde und Familienangehörige bei Ihnen zu Hause zu bewirten und zu unterhalten?

Keine	Wenig	Ziemlich	Stark	Wegen Sehkraft aufgehört	Aus anderen Gründen aufgehört oder kein Interesse
1	2	3	4	5	6

64. Haben Sie aufgrund Ihrer Sehkraft Schwierigkeiten, unter schlechten Bedingungen Auto zu fahren, wie bei schlechtem Wetter, in der Hauptverkehrszeit, auf der Autobahn oder im Stadtverkehr?

Keine	Wenig	Ziemlich	Stark	Wegen Sehkraft aufgehört	Aus anderen Gründen aufgehört oder kein Interesse
1	2	3	4	5	6

Die nächsten Fragen behandeln Dinge, die Sie vielleicht aufgrund Ihrer Sehkraft tun, z.B. bei der Arbeit oder bei täglichen Aktivitäten im Haushalt, bei der Kinderpflege, in der Schule oder bei gesellschaftlichen Aktivitäten. Bei jeder Frage antworten Sie bitte, ob dies für Sie immer zutrifft, meistens, manchmal, selten oder nie.

Immer	Meistens	Manchmal	Selten	Nie
1	2	3	4	5

65.	Benötigen Sie mehr Hilfe von anderen?	1	2	3	4	5
66.	Sind Sie in den Dingen, die Sie tun wollen, eingeschränkt?	1	2	3	4	5

Die nächsten Fragen behandeln Ihr Umgehen mit Ihrem Sehvermögen. Bitte markieren Sie für jede Aussage, ob sie für Sie absolut richtig ist, meistens richtig ist, ob Sie nicht sicher sind, ob sie meistens falsch ist oder absolut falsch ist.

Absolut richtig	Meistens richtig	Weiß nicht	Meistens falsch	Absolut falsch
1	2	3	4	5

67.	Wegen meiner Sehkraft bin ich oft gereizt.	1	2	3	4	5
68.	Wegen meiner Sehkraft gehe ich nicht alleine aus dem Haus.	1	2	3	4	5

69.	Hatten Sie zu Beginn Ihrer Erkrankung Symptome (Ja) oder wurde die Erkrankung zufällig (z.B. im Rahmen einer Kontrolluntersuchung) diagnostiziert (Nein)?	Ja	Nein

Hiermit bestätige ich, _____________, dass ich o.g. Fragen nach bestem Wissen und Gewissen ausgefüllt habe und dabei meine eigenen Antworten wiedergegeben habe.

____
Unterschrift, Ort, Datum
