# Supplementary material for: Exploring Non-Modifiable and Modifiable Determinants of Vision-Related Quality of Life in Central Serous Chorioretinopathy
Source: J Clin Med. 2024 Jul 25;13(15):4359. doi: 10.3390/jcm13154359 (PMC11313338; doi:10.3390/jcm13154359)
Supplement: Supplementary file 1 [file jcm-13-04359-s001.zip › Suppl. Table S1.pdf]

Suppl. Table S1

	**Modifiable**	**Non-Modifiable**
**Lifestyle Habits**	Stress Management	Age
	Sleep Quality and Duration	Gender
	Physical Activity	Genetic Markers
	Smoking	Ethnicity
	Hormonal Treatments	Family History
	Alcohol Consumption	
	Diet and Nutrition	
	Sun Exposure	
	Use of Corticosteroids	
	Occupational Stress Management	
	Reduction of Caffeine Intake	
	Hydration	
	Recreational Drug Use	
	Sedentary Lifestyle	
	Excessive Screen Time	
	Poor Posture	
	Overuse of Digital Devices	
**Health Conditions**	Control of Comorbidities (e.g., Cushing's Syndrome, gastrointestinal conditions, hypertension)	Presence of CSCR in Relatives
	Management of Obesity	
	Control of Diabetes	
	Regular Health Check-ups and Monitoring of Eye Health	
	Treatment of Gastroesophageal Reflux Disease (GERD)	
	Management of Cardiovascular Health	
	Treatment of Sleep Apnea	
	Management of Autoimmune Disorders	
	Control of High Blood Pressure	
	Management of Thyroid Disorders	
**Psychological Factors**	Type A Personality	
	Anxiety and Depression Management	
	Use of Psychotropic Medications	
	Stress-Reduction Techniques (e.g., mindfulness, yoga)	
	Regular Mental Health Check-ups	
	Counseling and Therapy	
**Environmental Factors**	Reduction of Exposure to Toxins and Pollutants	
	Occupational Stress Management	
	Living Conditions (e.g., noise, light pollution)	
	Air Quality	
	Exposure to Secondhand Smoke	
	Climate and Seasonal Changes	
	Work Environment Ergonomics	
**Medical Treatments**	Avoidance of Certain Medications (e.g., corticosteroids, decongestants)	
	Adherence to Medical Advice and Prescriptions	
	Use of Supplements (under medical supervision)	
	Early Treatment of Infections	
